# Assessment of psychosocial factors and distress in women having adjuvant endocrine therapy for breast cancer: the relationship among emotional distress and patient and treatment-related factors

**DOI:** 10.1186/s40064-016-2136-2

**Published:** 2016-04-19

**Authors:** Ozturk Ates, Cem Soylu, Taner Babacan, Furkan Sarici, Neyran Kertmen, Deborah Allen, Ali Riza Sever, Kadri Altundag

**Affiliations:** Department of Medical Oncology, Hacettepe University Cancer Institute, 06100 Sihhiye, Ankara, Turkey; Department of Psychology, Beytepe Campus, Hacettepe University, Ankara, Turkey; Department of Radiology, Maidstone and Tunbridge Wells NHS Trust, Maidstone, UK; Department of Radiology, Hacettepe University School of Medicine, Ankara, Turkey

**Keywords:** Breast cancer, Endocrine therapy, Emotional distress, Psychosocial factors, Social support, Self-esteem

## Abstract

**Purpose:**

The aims of this study were to comprehensively describe the psychosocial and medical characteristics of women who initiated tamoxifen or aromatase inhibitors for breast cancer and to compare levels of emotional distress according to their medical (tumor demographics, treatment type, treatment duration) and psychosocial (self-esteem, perceived social support, sociodemographic) characteristics.

**Methods:**

A total of 104 women currently receiving tamoxifen or aromatase inhibitors was recruited from outpatient clinics and they were asked to complete self-report questionnaires including the Rosenberg Self-Esteem Scale, the Multidimensional Scale of Perceived Social Support and the Hospital Anxiety and Depression Scale during their routine follow-up. Psychosocial and medical characteristics of the patients were first described and subsequently the score of emotional distress was compared with these.

**Results:**

The patients’ mean age was 52.49 ± 10.30 and they were being treated for an average of 24.3 months. Out of the patients’ characteristics, educational and marital status, level of perceived social support and self-esteem were all significantly related with emotional distress. As for medical variables, the score of distress was relatively higher among patients in the first 2 years of their treatment than the patients who were in the second to fifth years of treatment, but this was not statistically significant.

**Conclusions:**

Given the results of this study, it appeared that patient variables, rather than the medical or treatment characteristics, were related with emotional distress in women undergoing endocrine treatment. For that reason it is critical that medical staff are aware of patient factors that relate to distress during a long period of adjuvant endocrine therapy.

## Background

Breast cancer is a major public health problem that affects women in many parts of the world as well as Turkey (Ferlay et al. 2015). Breast cancer treatments have substantially improved over the past two decades, which have led to a reduction in breast cancer mortality due to both screening and improvements in therapy (Berry et al. 2005).

Approximately 70 % of all breast cancers are hormone receptor positive (Harvey et al. 1999) and in this group of patients, upon completion of primary therapy (e.g., surgery, radiation, chemotherapy), adjuvant endocrine therapy (AET) with either tamoxifen or aromatase inhibitors (AIs) is an important component (Early Breast Cancer Trialists’ Collaborative Group 2005). The widely accepted standard for AET for hormone receptor positive breast lesions for many years was the administration of tamoxifen for the duration of 5 years (Howell et al. 2005). However with the development of the third-generation AIs (i.e. anastrozole, letrozole and exemestane) the use of tamoxifen was replaced in postmenopausal patients (Smith and Dowsett 2003). A lot of studies indicated that both regimens, tamoxifen and AIs, are appropriate treatment options in these patients as they reduce risk of disease relapse and death (Burstein et al. 2014; Regan et al. 2011).

Although AET can improve disease-free survival in breast cancer patients, some studies indicated that it is associated with considerable side effects that negatively impact women’s well-being (von Blanckenburg et al. 2013). During AET, many breast cancer patients can experience a range of physical and psychological side effects such as hot flushes, joint pain and emotional distress (ED) (Rosenberg et al. 2015; van Londen et al. 2014; Bower 2008). Distress is a multifactorial unpleasant emotional experience of a psychological, social and/or spiritual nature that may interfere with the ability to cope effectively with cancer, its symptoms and its physical treatment (National Comprehensive Cancer Network 2011). While most of the previous studies focused on the physical adverse effects in women taking AET, very few studies addressed the emotional problems such as anxiety and depression among these patients (Rosenberg et al. 2015). From these limited studies, Ploos van Amstel et al. 2013 conducted research among breast cancer survivors of whom 37 % were still treated with hormonal therapy and showed that one-third of all cancer survivors experienced distress. However in a different study, it was reported that depression scores were within the normal range in patients treated with tamoxifen or raloxifene in comparison to healthy women in similar age group (Land et al. 2006). More specifically, Schilder et al. (2009) evaluated symptoms of depression in breast cancer patients treated with tamoxifen or exemestane and healthy control groups and researchers indicated that there were no differences between patients and control groups with regard to mental health or depressive symptoms.

The objectives of our cross-sectional study were to describe comprehensively the psychosocial characteristics of patients and to investigate the relationship between psychosocial and medical characteristics and self-reported ED in women with breast cancer who are on tamoxifen or aromatase inhibitors. Knowledge about this relationship is fundamental to develop necessary interventions to reduce ED in women during the long treatment journey. Psychosocial factors were assessed by evaluating self-esteem, perceived social support and patients’ sociodemographic characteristics including age, marital status, number of children, level of education, socioeconomic status, body mass index and menopausal status at diagnosis. Illness and treatment-related factors were assessed by evaluating a broad range of medical factors such as type and duration of AET, type of surgery, tumor stage and grade, and administration of chemotherapy or radiotherapy. Most of the included medical and psychosocial parameters in this study have not been investigated in previous studies.

## Methods

### Patients

We employed a cross-sectional, descriptive, and correlational design. Participants were recruited consecutively between September 2015 and December 2015 during their routine outpatient clinic visits in Hacettepe University, Institute of Oncology. Patients were considered eligible for participation if they were diagnosed with non-metastatic (stages I–III) hormone receptor-positive breast cancer, had completed their surgical treatment, chemotherapy, and/or radiation therapy and were currently taking adjuvant endocrine therapy (tamoxifen or AIs) for at least 1 month. All included patients were female, older than 18 years of age and had graduated from primary school or higher. Patients were excluded if they were taking AET as part of treatment for metastatic disease or had documented psychiatric or neurological disorders that could interfere with study participation (e.g., dementia or schizophrenia).

Of the 130 women approached, 87 % agreed to participate. A further nine agreed participants had to be excluded due to the following reasons: 4 women took AET for less than 1 month and 5 women did not complete the study questionnaire despite their consent. 104 women were included in the final analysis.

### Study protocol

The study was approved by the ethical committee of the Hacettepe University, Faculty of Medicine. The researcher and medical oncologists read relevant medical records to search for potential patients in order to decide patients’ eligibility for this study. Patients who passed this pre-screening were informed and the purpose of the study was explained to them by their oncologists at the time of routine medical follow-up. When patients agreed to participate in the study, they were contacted by the study research assistant who provided further information. They were informed that their involvement was voluntary and they could withdraw from the study any time without affecting their care. After written and verbal informed consent was obtained from each participant, data regarding their age, educational and marital status, number of children, and socioeconomic status was asked during face-to-face interviews using a demographic questionnaire. Subsequently, patients completed self-report questionnaires. If possible, we requested patients to complete the questionnaire without assistance and support was given only when it was necessary. The medical information including tumor stage and grade, menopausal status, type of surgery, type and duration of AET, and administration of chemotherapy and/or radiotherapy was obtained from medical records.

## Questionnaires

### Demographic and Clinical Questionnaire

The sociodemographic information of the patients is listed in Table 1 and, as indicated above, was obtained by interview, while cancer and treatment related data was abstracted from the patients’ medical charts. Patients’ height and weight measurements were collected from medical charts and used to calculate body mass index (BMI). BMI was calculated by dividing body weight (in kilograms) by the square of height in meters (kg/m^2^).

**Table 1:** Socio-demographic and medical characteristics of patients (*n* = 104)

Patient factors	% (*n*)	Cancer and treatment factors	% (*n*)
Age [mean (min–max)]	52.49 (32–77)	Time (months) since AET initiation	24.38 (1–58)
Marital status		Between 1 and 24 months	49 (51)
Married	75.9 (79)	Between 25 and 58 months	51 (53)
Single^a^	24 (25)	Current treatment	
Children		Tamoxifen^b^	47.1 (49)
One or more	82.7 (86)	Aromatase inhibitors (AIs)^c^	52.9 (55)
None	17.3 (18)	Radiotherapy	
Educational level		Yes	71.2 (74)
Primary or secondary school	33.7 (35)	No	28.8 (30)
High school	32.7 (34)	Chemotherapy	
College or above	33.7 (35)	Yes	61.5 (64)
Socioeconomic status		No	38.5 (40)
Low	17.3 (18)	Pathological tumor stage	
Middle	70.2 (73)	T1	40.4 (42)
High	12.5 (13)	T2	51 (53)
Body mass index (kg/m^2^)		T3	8.7 (9)
≤ 24.9	31.7 (33)	Histological grade	
25–29.9	40.4 (42)	Grade I	11.5 (12)
≥30	27.9 (29)	Grade II	49 (51)
Menopausal status		Grade III	39.4 (41)
Pre-menopausal	46.2 (48)	Type of surgery	
Peri or post-menopausal	53.8 (56)	Mastectomy	56.7 (59)
		Breast conserving surgery	43.3 (45)

^a^Includes single, divorced, separated, widower

^b^2 patients used aromatase inhibitors then switched tamoxifen

^c^AIs includes anastrozole and letrozole; 4 patients used tamoxifen then switched to AIs

### Hospital Anxiety and Depression Scale (HADs)

This is a self-reported scale originally developed to indicate the possible presence of anxiety and depression states (Zigmond and Snaith 1983). It contains two subscales (one for anxiety and the one for depression) and 14 items. Total scores for each subscale range from 0 to 21, and higher scores represent greater anxiety or depression. The Turkish version of this scale was assessed by Aydemir et al. (1997) for its validity and reliability (Aydemir et al. 1997). Aydemir et al. (1997) stated that the Cronbach alpha was 0.85 and 0.77 for anxiety and depression subscales respectively. In this study the Cronbach alpha was 0.84 in the anxiety subscale and 0.80 in the depression subscale.

While the HADs scale is accepted as valid and reliable in cancer patients and is used frequently as a standard in the field of clinical oncology/psychology services (Vodermaier and Millman 2011), recent studies have indicated that it does not provide a good separation between symptoms of anxiety and depression (Norton et al. 2013). However it is effective in measuring the level of emotional distress (Norton et al. 2013; Cosco et al. 2012). For that reason we decided to use the ‘HADs Total’ in emotional distress calculations.

### Multidimensional Scale of Perceived Social Support (MSPSS)

The MSPSS was used to measure the level of perceived social support from family, friends, and significant others. It contains 12 items and each item is rated on a 7 point scale. (Zimet et al. 1988). All items are added and then divided by 12 for the total score. High scores indicate a high degree of perceived social support. Its validity and reliability in Turkey was performed by Eker and Arkar (1995) and the Cronbach alpha was 0.89 (Eker and Arkar 1995). In this this study the Cronbach alpha was found to be 0.88.

### Rosenberg Self-Esteem Scale (RSES)

This scale measures global self-esteem with a 10-item scale based on direct self-reporting (Rosenberg 1965). Possible scores of the scale range from 10 to 40. Higher scores represent more positive attitudes toward the self. This scale was adapted to the Turkish population by Cuhadaroglu (1986) and the correlation between the scale and psychiatric interview results was found to be 0.71 and the test–retest reliability was reported as 0.75 (Cuhadaroglu 1986). For the present study, the Cronbach alpha of the scale was 0.85.

### Statistical analysis

Based on the previous studies, we divided the total patients into two groups: Group one comprised patients having AET for a duration of between 1 and 24 months and group two comprised patients on AET between 25 and 60 months. Such a division was performed as some researchers have indicated that breast cancer survivors experience more distress in the first 2 years than in the period between 2 and 5 years (Ploos van Amstel et al. 2013). Therefore, we expected that ED might be higher during the first 2 years of AET compared to the second period.

All data was analyzed using SPSS software version 22 for Windows. The patients’ sociodemographic and clinical characteristics were summarized with means, standard deviations, ranges, and frequencies in order to provide a description of the sample of patients. The associations between sociodemographic and clinical variables were assessed using the Mann–Whitney U test and Kruskal–Wallis statistics on the data distribution. In cases of a significant difference among three groups, the Post Hoc Mann–Whitney U test was used to compare the three groups on dependent variables, using a Bonferonni corrected p value of 0.017 to indicate statistical significance. Relationships between ED, self-esteem, social support and age were examined by the Spearman product moment correlation coefficients.

## Results

### Patient characteristics

Socio-demographic and medical characteristics of the patients are listed in Table 1. The mean age was 52.49 (SD ± 10.30) years. Seventy-nine (75.9 %) patients were married, and 86 (82.7 %) had one or more children. Approximately two-thirds of women (66.4 %) had been educated at high school level or above. The majority of participants (70.2 %) reported their socio-economic status as middle class. Roughly 70 % of participants had a BMI within the overweight or obesity category and more than half of women (53.8 %) were peri- or post-menopausal. With regards to treatment, the duration of AET for the overall sample ranged from 1 to 58 months (Mean = 24.38, SD = 18.26). More than half were taking AIs (52.9 %), and the remaining women were taking tamoxifen (47.1 %) at the time of participation in this study. Before AET treatment, 71.2 % of women received radiotherapy and 61.5 % received chemotherapy. With regards to the primary breast tumor and grade, nearly half of the patients were reported as T2 and grade II (51 and 49 % respectively). Fifty-nine patients (56.7 %) underwent mastectomy, while the remainder had breast-conserving surgery.

### Relationship between emotional distress and patients’ socio-demographic and clinical factors

Table 2 reveals that, with regards to the level of ED, unmarried women were significantly different from married women. Analysis of the two groups’ means indicated that the average ED score for single women (19.12 ± 10.47) was significantly higher than the score for married women (13.17 ± 7.87). A Kruskal–Wallis analysis of variance indicated that the three patients’ education groups differed significantly on ED χ^2^(2, *N* = 104) = 9.49, p = 0.009. Post Hoc Mann–Whitney tests (using a Bonferonni corrected p value of 0.017 to indicate statistical significance) compared the three patients’ education groups. According to this analysis, women who completed primary or secondary school graduates (18.68 ± 9.57) had significantly higher ED compared to women who were high school graduates (12.38 ± 7.23) and those who had graduated from college or above (12.68 ± 8.43). Significant associations were not observed between ED and other patient characteristics (number of children, body mass index, menopausal status, and socioeconomic status) and the medical variables of patients (type of current treatment, duration category of AET used, received radiotherapy and chemotherapy, tumor stage, histological grade).

**Table 2:** Distributions of level of psychological distress according to medical and sociodemographic characteristics of patients

Patient characteristics	% (*n*)	HAD total mean ± SE	Treatment factors	*n* (%)	HAD total mean ± SE
Marital status			Current Treatment		
Married	75.9 (79)	13.17 ± 7.87	Tamoxifen	47.1 (49)	13.65 ± 8.30
Single	24 (25)	19.12 ± 10.47	Aromatase inhibitors (AI)	52.9 (55)	15.45 ± 9.37
		MWU = 704, p = 0.031			MWU = 1223, p = 0.417
Children			Duration of AET		
One or More	82.7 (86)	14.36 ± 8.53	Between 1 and 24 months	49 (51)	16.01 ± 9.19
None	17.3 (18)	15.77 ± 10.60	Between 25 and 58 months	51 (53)	13.24 ± 8.45
		MWU = 761, p = 0.911			MWU = 1073, p = 0.070
Education			Pathological tumor stage		
Primary or secondary	33.7 (35)	18.68 ± 9.57	T1	40.4 (42)	15.40 ± 9.28
High school	32.7 (34)	12.38 ± 7.23	T2	51 (53)	14.30 ± 8.83
College or above	33.7 (35)	12.68 ± 8.43	T3	8.7 (9)	12.66 ± 7.77
		KW = 9.489, p = 0.009			KW = 0.589, p = 0.745
Body mass index			Histological grade		
≤24.99	31.7 (33)	12.48 ± 7.12	Grade I	11.5 (12)	12.91 ± 9.11
25–29.9	40.4 (42)	14.33 ± 9.65	Grade II	49 (51)	15.60 ± 8.98
≥30	27.9 (29)	17.41 ± 9.08	Grade III	39.4 (41)	13.85 ± 8.77
		KW = 4.188, p = 0.123			KW = 1.750, p = 0.417
Menopausal status			Type of surgery		
Pre-menopausal	46.2 (48)	13.50 ± 8.43	Mastectomy	56.7 (59)	14.66 ± 8.76
Peri or post-menopausal	53.8 (56)	15.55 ± 9.23	Breast conserving surgery	43.3 (45)	14.53 ± 9.14
		MWU = 1194, p = 0.329			MWU = 1308, p = 0.901
Socioeconomic status			Radiotherapy		
Low	17.3 (18)	16.05 ± 10.11	Yes/no	71.2 (74)/28.8 (30)	14.97 ± 8.97/13.70 ± 8.75
Middle	70.2 (73)	14.24 ± 8.69			MWU = 1020, p = 0.520
High	12.5 (13)	14.61 ± 8.73	Chemotherapy		
		KW = 0.512, p = 0.774	Yes/no	61.5 (64)/38.5 (40)	15.21 ± 9.02/13.62 ± 8.69
					MWU = 1129, p = 0.314

*MWU* Mann–Whitney U, *KW* Kruskall Wallis

### Relationship between self-esteem, perceived social support and emotional distress

As indicated in Table 3, ED was negatively correlated with perceived social support (*r* = −0.32, p < 0.01) and self-esteem (*r* = −0.35, p < 0.01). This means that having greater perceived social support and self-esteem was related to lower ED. The age of patients was not related with ED (*r* = 0.43, p > 0.05).

**Table 3:** Means, SDs, and correlations among study variables

	Mean	SD	1	2	3	4
1. Psychological distress	14.60	8.88	–			
2. Perceived social support	58.84	13.21	−0.32*	–		
3. Self esteem	25.76	7.49	−0.35*	0.38*	–	
4. Age of patients	52.49	10.30	0.043**	−0.16*	–0.13*	–

* p < 0.01; ** p > 0.05

## Discussion

After the completion of primary treatment, patients with breast cancer often receive long-term AET to reduce disease recurrence and to improve survival; therefore, awareness about their psychological experiences is important in this long duration of treatment. For that reason, we designed the current study to provide a more detailed picture of the psychosocial characteristics of women undergoing AET and focused on the relationship between these factors and levels of ED.

Mehnert and Koch (2008) indicated that lower education is a predictor of psychological comorbidity in patients with breast cancer. Conversely Gómez-Campelo et al. 2014 reported that having a higher educational level was associated with more psychological distress. In our study, in line with Menhert and Koch, we found that patients who completed primary or secondary school were significantly more likely to have a higher level of ED than those who had completed high school and college or above. Our findings might be explained by the fact that patients with higher educational levels may have more awareness about recent advances in treatment options from different sources such as the Internet, and as a result have less psychological distress. However more research is needed to elucidate the effect of education on ED. In addition to level of education, we found that single patients reported more distress than those who were married. It can be speculated that married women may have more physical support from their spouses or children compared to those who are single. Physical support may be important in dealing with problems such as transportation, particularly when patients are having aggressive treatment. Married women may also be less exposed to stressful life situations than single women due to their greater financial power. Therefore, unmarried patients might have experienced higher ED levels. Consistent with our study, Giese-Davis et al. 2012 indicated that unmarried women are particularly vulnerable to experiencing practical and psychosocial problems while being married protects against such problems.

In previous studies, some researchers indicated that anxiety and depressive symptoms were not significantly affected by endocrine therapy during the first 3 years of treatment (Nystedt et al. 2003). In a different study, Land et al. 2006 investigated symptoms of depression over a period of 60 months and demonstrated that the depression score remained unchanged during the entire course of treatment (Land et al. 2006). In our study, while the difference was not at the statistical level, we found that women who were taking AET revealed higher ED in their first 2 years of treatment compared to those who were within the 25th to 60th months. This difference of lower ED levels at the late (3- to 5-year) period may be secondary to the fact that more time has elapsed since the initial aggressive treatments (i.e. surgery, chemo and radiotherapy). Future research is required to clarify the relationship between ED and duration of AET. In addition to these findings, in line with previous studies, our study showed no significant difference in ED score between patients treated with tamoxifen and those who were treated with AIs (Schilder et al. 2009; Takei et al. 2012; Ohsumi et al. 2011).

Perceived social support has been established as protective factor, which mitigates psychological distress in breast cancer patients (Friedman et al. 2006). In accordance with that study, our data revealed that perceived social support and self-esteem were negatively related with emotional distress. These findings also support the results of Brunet et al. (2014) that perceived support could be one way to help patients to cope with stress. In the light of these studies, medical staff should identify patients who have low perceived social support and self-esteem and refer them to psycho-oncology units.

### Limitations

Our study has some limitations. It has a cross-sectional design which provides no information on causal relationship. For that reason, longitudinal studies are required to establish more clearly the causal relationship between distress, patient related factors and medical characteristics and future studies are also needed to examine mediating/moderating effects of the identified risk factors. In addition, we did not evaluate the data regarding the comorbidities of patients. Despite these limitations, our study is noteworthy because we broadly assessed the range of factors that related with distress along with perceived social support and self-esteem.

### Implications for clinical practice

Patient-reported outcomes are particularly useful in the setting of prevention. In this respect, outcomes of the present study will assist in the design of interventions to prevent or manage the psychological mood of patients with breast cancer on AET. The current findings suggest that professionals need to continuously assess and monitor depressive and anxiety symptoms in breast cancer patients after initiation of AET especially if they factors related to high levels of ED as described above. As several recent studies have already identified that high distress is a risk factor for poorer medical adherence of AET (Cahir et al. 2015; Colleoni et al. 2000), our study results are of significance to ensure the continuation of treatment by detecting potential patients who may be prone to have higher distress in a timely fashion.
